# Supplementary material for: An organic/inorganic electrode-based hydronium-ion battery
Source: Nat Commun. 2020 Feb 19;11:959. doi: 10.1038/s41467-020-14748-5 (PMC7031366; doi:10.1038/s41467-020-14748-5)
Supplement: Supplementary file 1 — Supplementary Information [file 41467_2020_14748_MOESM1_ESM.pdf]

An organic/inorganic electrode-based hydronium-ion battery

Guo et al.

Supplementary Figures

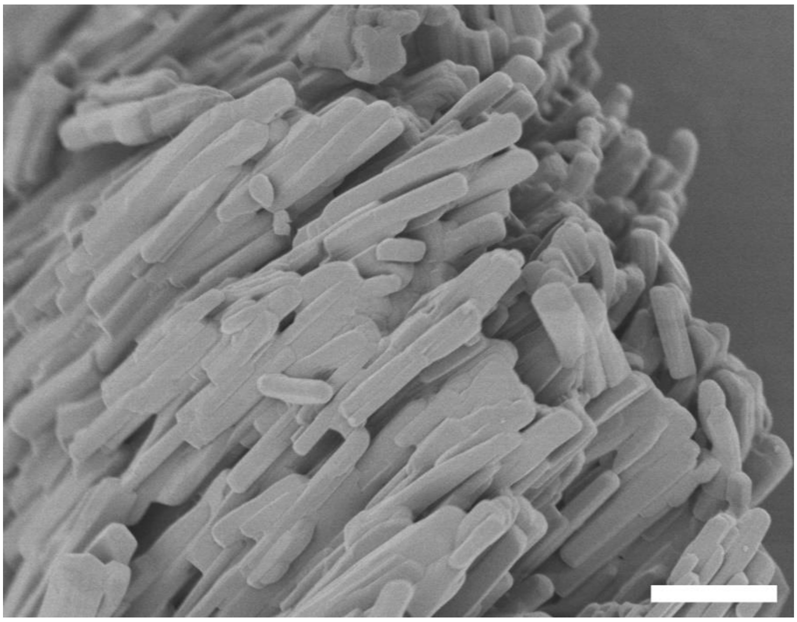

Supplementary Figure 1 | Scanning electron microscope (SEM) image of as-prepared PTO particles. Scale bar: 2 μm.

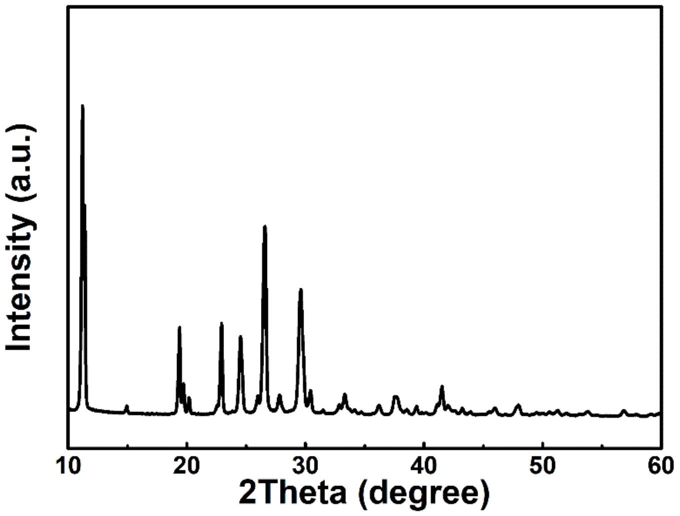

Supplementary Figure 2 | X-ray diffraction (XRD) patterns of as-prepared PTO material.

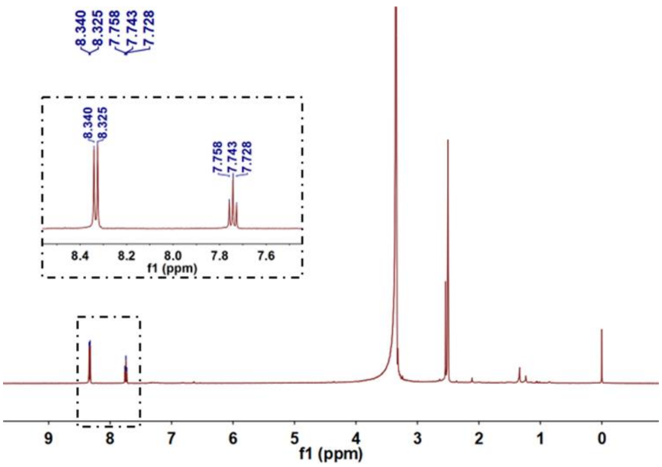

Supplementary Figure 3 | ^{1}H Nuclear magnetic resonance (^{1}H NMR) spectrum (500 MHz, DMSO-d6) of as-prepared PTO material.

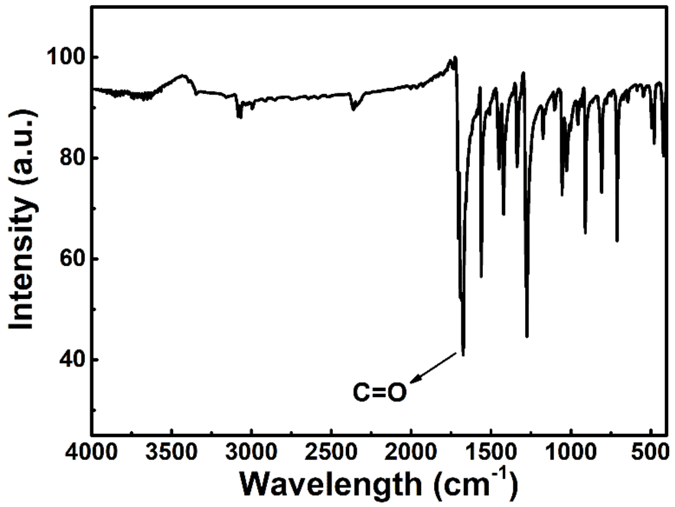

Supplementary Figure 4 | Ex-situ Fourier transform infrared spectroscopy (FT-IR) spectra of as-prepared PTO material. The characteristic peak at ~1670 cm^{-1} is ascribed to carbonyl groups on PTO molecules.

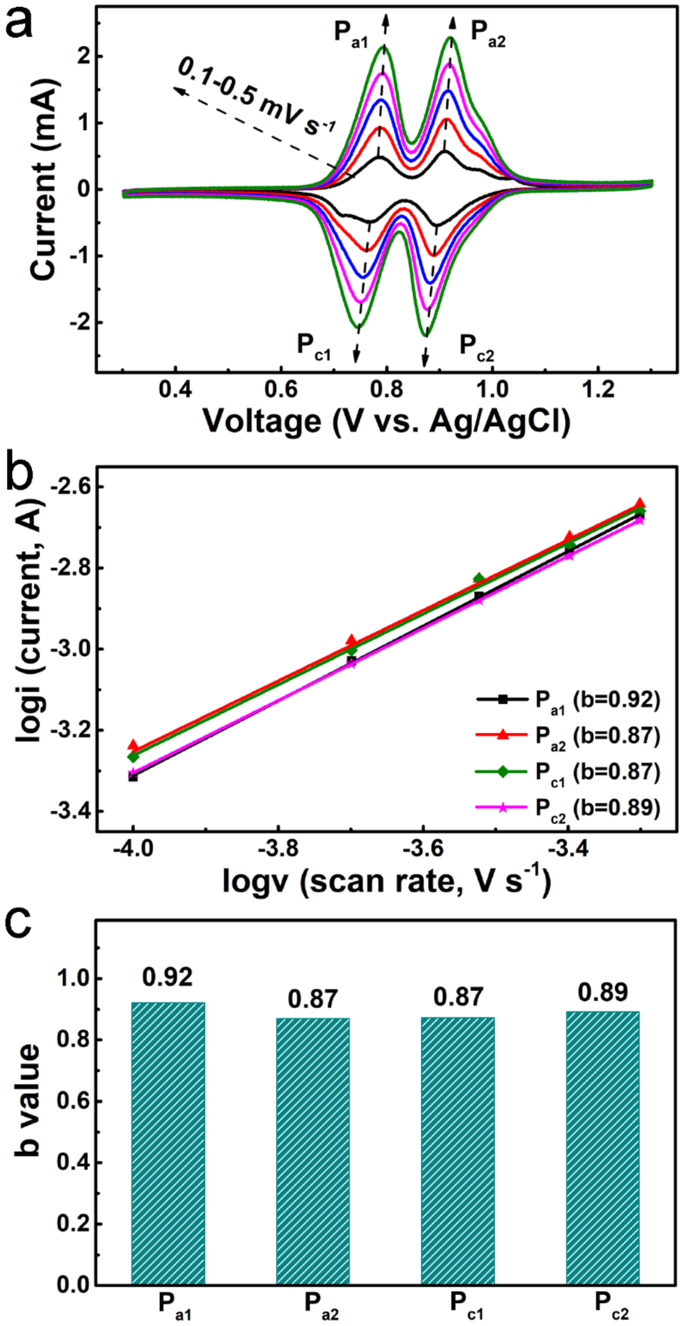

Supplementary Figure 5 | ** Kinetics property investigations of the H_3O^+ storage behavior of the PTO-electrode. **a, CV curves at various scan rates from 0.1 to 0.5 mV s^{-1} of the PTO-electrode. **b**, log(i) ~ log(v) plots of anodic and cathodic peaks derived from above CV curves. **c**, b values of anodic and cathodic peaks (P_{a1}, P_{a2}, P_{c1}, P_{c2}) as 0.92, 0.87, 0.87 and 0.89, respectively. 2 M $MnSO_4$ + 2 M H_2SO_4 solution was used as electrolyte. [Generally, the relationship between the peak currents (*i*, A) and scan rates (*v*, V s^{-1}) can be described as $i = av^b$, where *a* and *b* are constants. That the coefficient *b*, the slope of log(i) ~ log(v) plots, close to 1.0 indicates a high rate behavior of the PTO-electrode.]

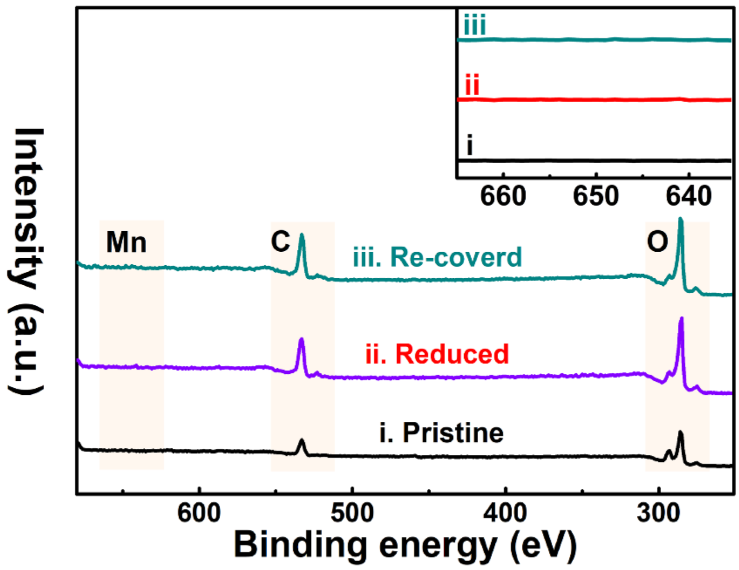

Supplementary Figure 6 | XPS spectra of PTO electrodes at various charged/discharged states. PTO electrodes were tested at pristine (i), fully-reduced (ii), and re-covered states (iii). The insert displays amplified XPS spectra of Mn element. The PTO-electrode was tested at a current density of 2 mA cm^{-2} in a three-electrode system for examination, using 2 M $MnSO_4$ + 2 M H_2SO_4 as electrolyte.

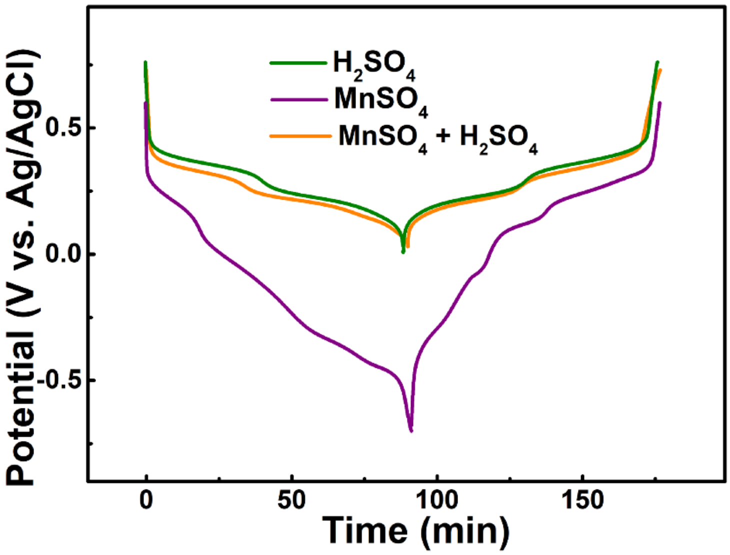

Supplementary Figure 7 | The galvanostatic discharge-charge profiles of PTO-electrode in different electrolytes (i.e. 2 M $MnSO_4$ + 2 M H_2SO_4, 2 M $MnSO_4$ and 2 M H_2SO_4), tested at a current density of 2 mA cm^{-2} in a three-electrode system using AC electrode and Ag/AgCl electrode as counter and reference electrodes, respectively.

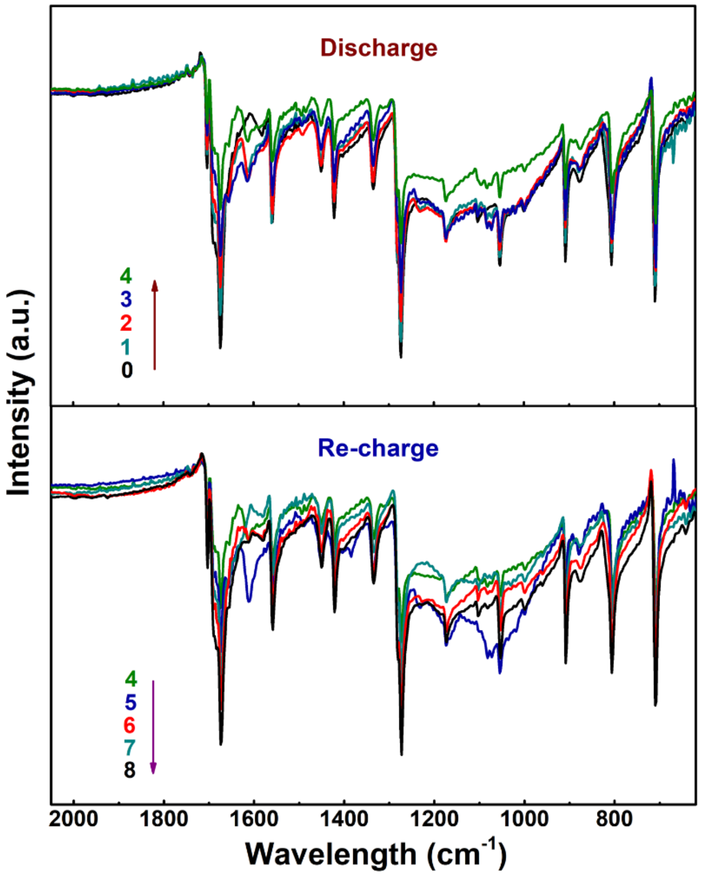

Supplementary Figure 8 | Ex-situ FT-IR spectra of PTO-electrode at various discharged-charged states during a discharge-charge cycle. PTO-electrodes underwent being discharged to 0 state (pristine), 1 state (discharged to 0.43 V), 2 state (discharged to 0.35 V), 3 state (discharged to 0.27 V), 4 state (discharged to 0 V) and then being re-charged to 5 state (re-charged to 0.30 V), 6 state (re-charged to 0.37 V), 7 state (re-charged to 0.45 V), 8 state (fully-charged to 0.7 V). All the discharge-charge experiments were acquired at a current density of 2 mA cm^{-2} in a three-electrode system, with MnO_2@GF electrode and Ag/AgCl electrode as counter and reference electrodes, respectively, using 2 M $MnSO_4$ + 2 M H_2SO_4 hybrid solution as electrolyte. It should be noted that the FT-IR spectra were manipulated by a routine baseline correction and background removal process for the clear observation of characteristic absorptions.

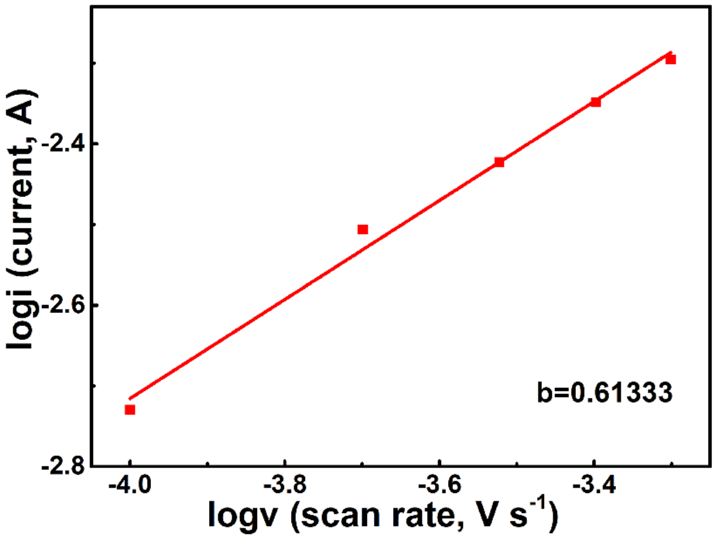

Supplementary Figure 9 | log(i) ~ log(v) plots of cathodic peaks of GF-electrode derived from CV curves in **Figure 3a**. The GF-electrode was tested in a three-electrode system with AC electrode and Ag/AgCl electrode as counter and reference electrodes, respectively. 2 M $MnSO_4$ + 2 M H_2SO_4 solution was used as electrolyte. [b value of the cathodic peaks, the slope of log(i) ~ log(v) plots, is calculated as about 0.61.]

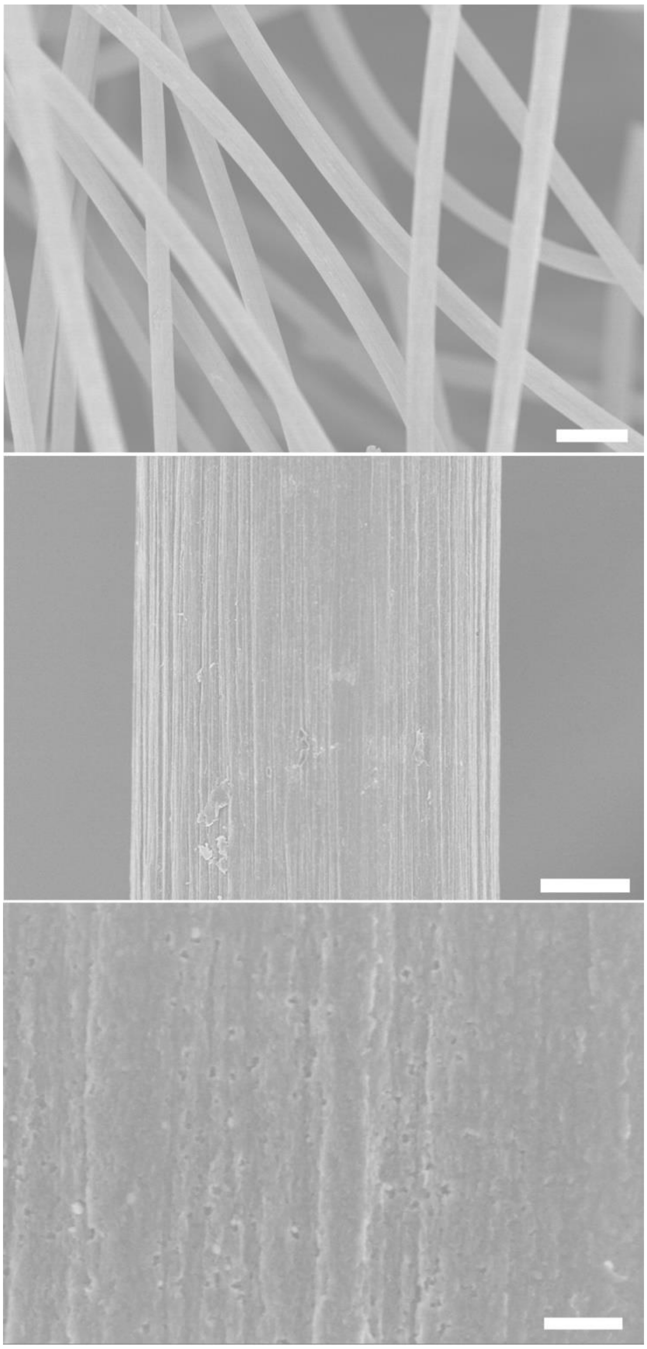

**Supplementary Figure 10 | **SEM images of the pristine GF-electrode. Scale bar: 20 μm (top), 2 μm (middle) and 200 nm (bottom). [It can be observed from the above SEM images that the GF electrode is composed with plenty of carbon fibers (i.e. GF fibers).]

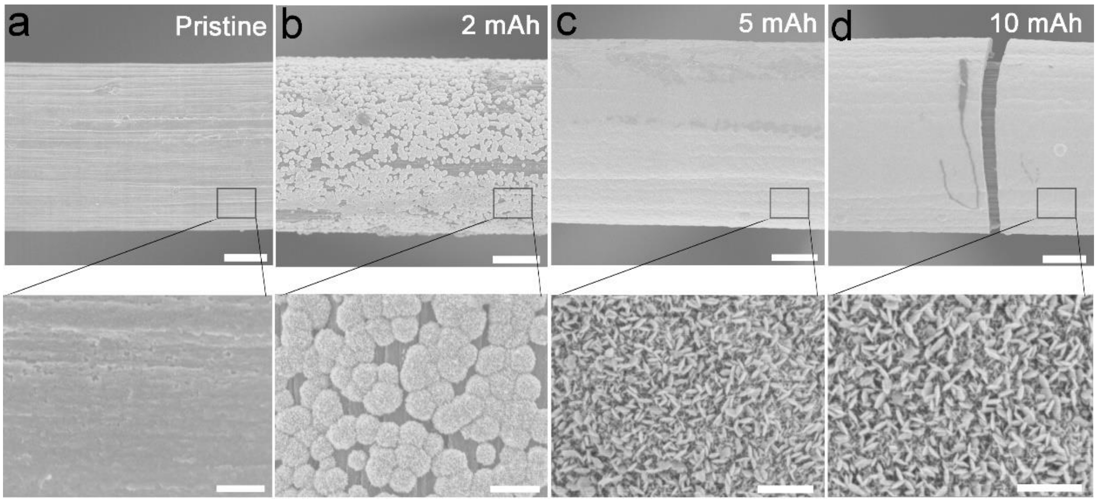

Supplementary Figure 11 | ** SEM images of GF-electrode at pristine and various charged states. GF electrodes were observed at pristine (a**), 2 mAh MnO_2 (**b**), 5 mAh MnO_2 (**c**) , and 10 mAh MnO_2 (**d**) electrodeposition situations under a constant deposition current of 5 mA (top, scale bar: 2 μm) and corresponding zoom-in images (bottom, scale bar: 200 nm). A three-electrode system was used to electrodeposit MnO_2, with AC electrode and Ag/AgCl electrode as counter and reference electrodes, respectively. 2 M $MnSO_4$ + 2 M H_2SO_4 solution was used as electrolyte.

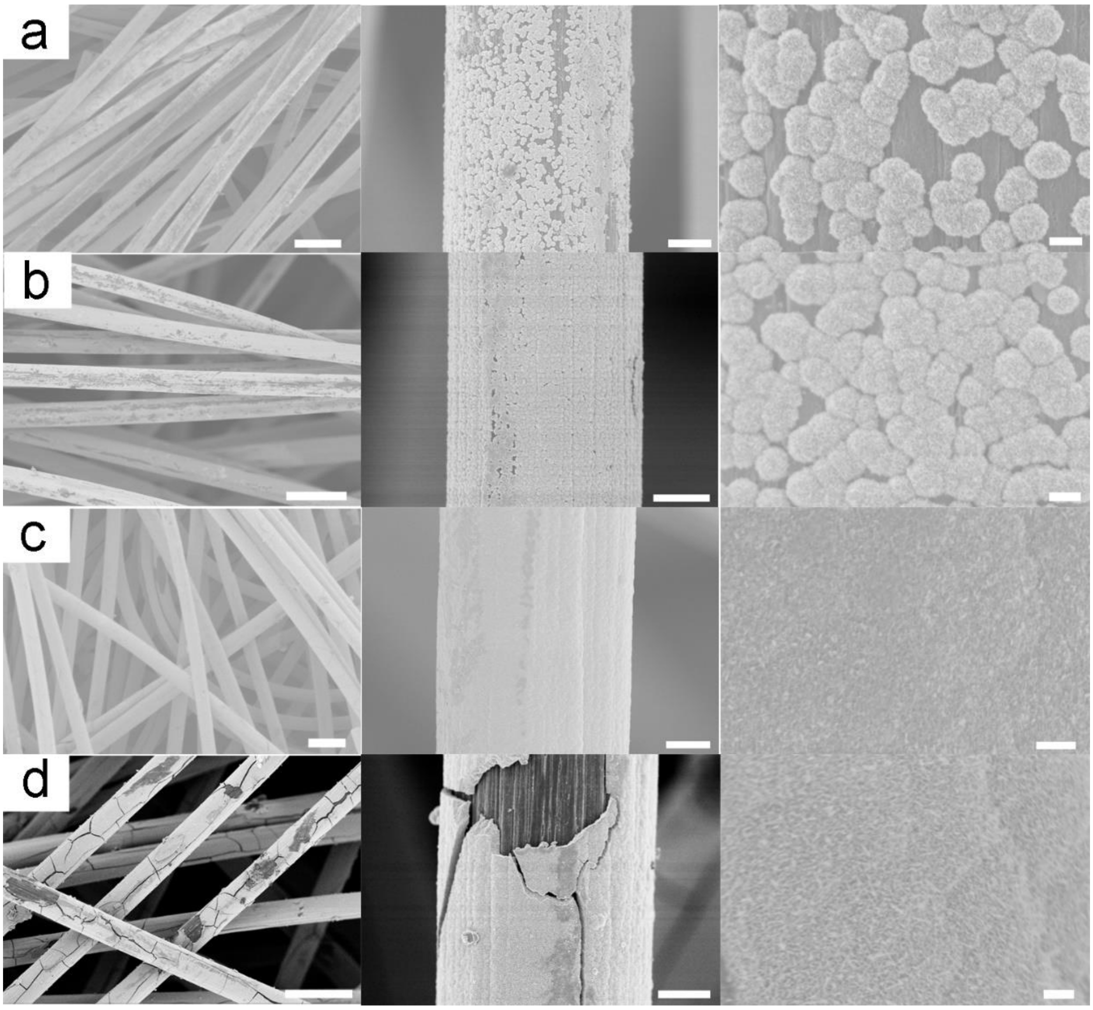

Supplementary Figure 12 | ** Morphology investigations by SEM images of MnO_2@GF cathode undergoing various MnO_2 electrodeposition capacities on GF. GF electrodes were charged for 0.2 hr (a**), 0.4 hr (**b**), 1 hr (**c**), and 2 hrs (**d**), respectively, at a constant current density of 5 mA cm^{-2}. Scale bar: 20 μm (left), 2 μm (middle) and 200 nm (right). A three-electrode system was used to electrodeposit MnO_2, with AC electrode and Ag/AgCl electrode as counter and reference electrodes, respectively. 2 M $MnSO_4$ + 2 M H_2SO_4 solution was used as electrolyte.

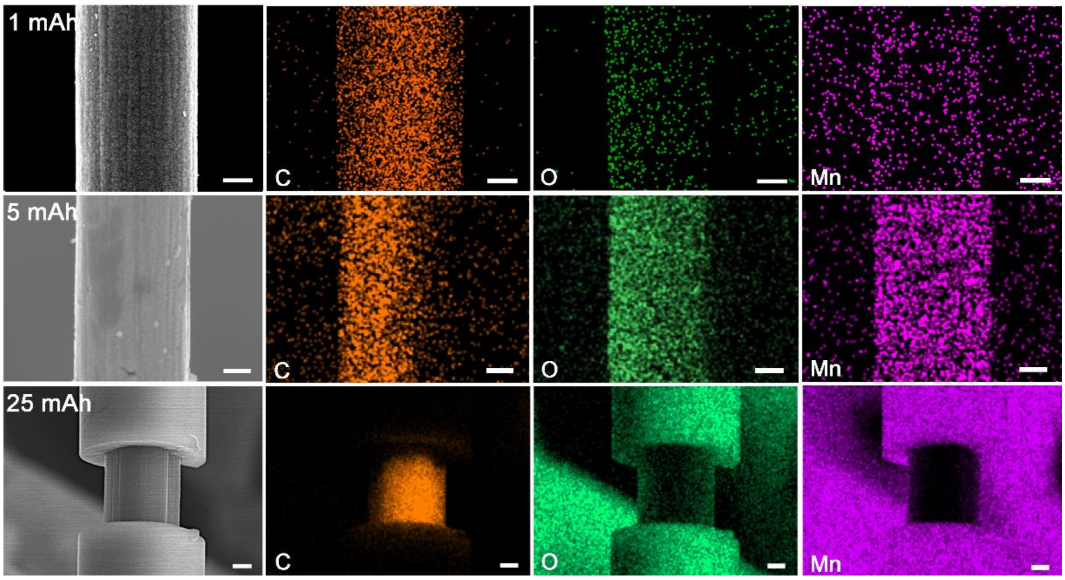

Supplementary Figure 13 | SEM images and EDS-elemental mapping images of GF-based electrodes with various MnO_2 deposition capacities of 1 mAh cm^{-2} (with a current of 2 mA cm^{-2}), 5 mAh cm^{-2} (with a current of 10 mA cm^{-2}), and 25 mAh cm^{-2} (with a current of 50 mA cm^{-2}). Scale bar: 2 μm. [It can be detected that MnO_2 layer was uniformly distributed on GF surface with various applied currents and deposition capacities.]

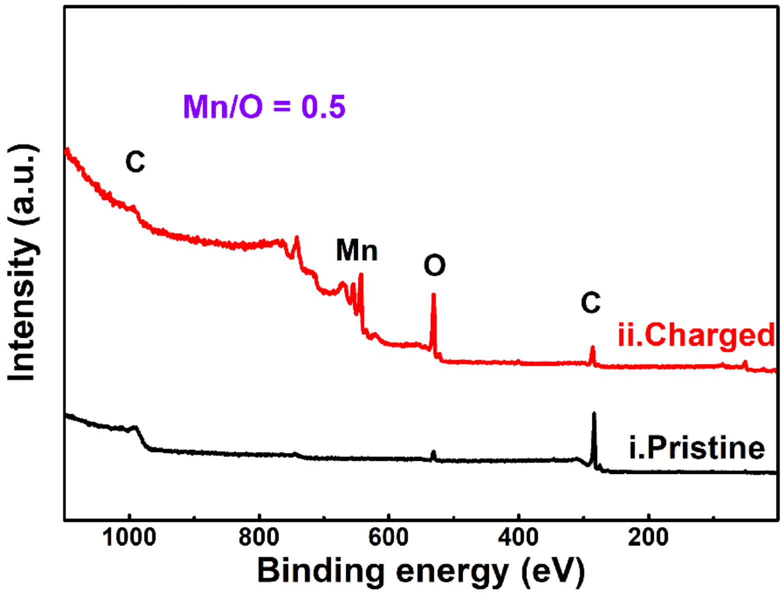

Supplementary Figure 14 | XPS spectra of GF-electrode at various states. (i) At pristine, and (ii) charged states. The GF-electrode was charged at a current density of 2 mA cm^{-2} in a three-electrode system for examination, using AC electrode and Ag/AgCl electrode as counter and reference electrodes, respectively. 2 M $MnSO_4$ + 2 M H_2SO_4 was used as electrolyte.

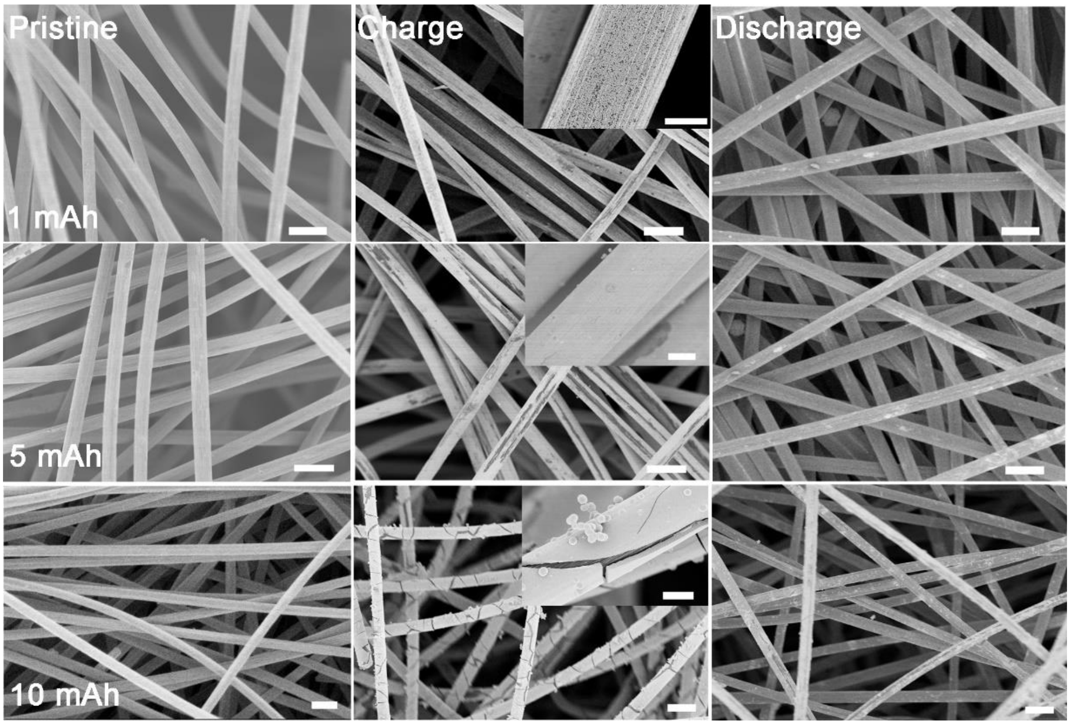

Supplementary Figure 15 | SEM images for GF electrodes at various charged/discharged states. GF electrode morphology was observed at pristine, charged (1 mAh, 5 mAh, 10 mAh MnO_2 deposition capacities), and discharged states with a current density of 5 mA cm^{-2}. Scale bar: 20 μm (5 μm for the insert).

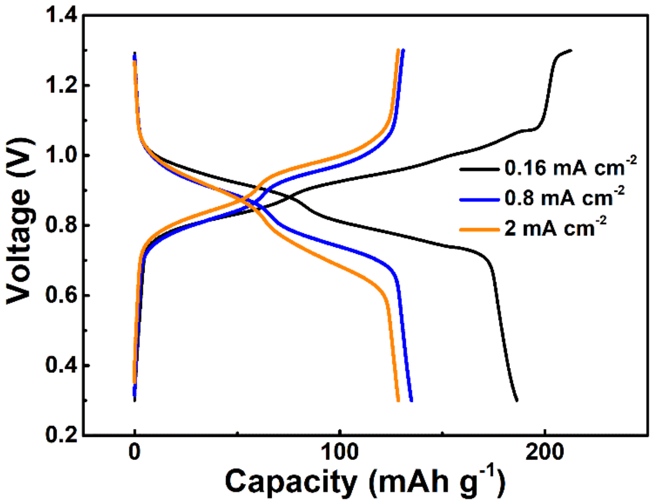

Supplementary Figure 16 | Galvanostatic charge-discharge curves of PTO//MnO_2@GF hydronium-ion battery with a very high mass loading of 25 mg cm^{-2} PTO in anode at various current densities of 0.16 mA cm^{-2}, 0.8 mA cm^{-2}, and 2 mA cm^{-2}. 2 M $MnSO_4$ + 2 M H_2SO_4 was used as electrolyte. The active electrode areas of PTO anode and MnO_2@GF cathode are all 1 cm^2.

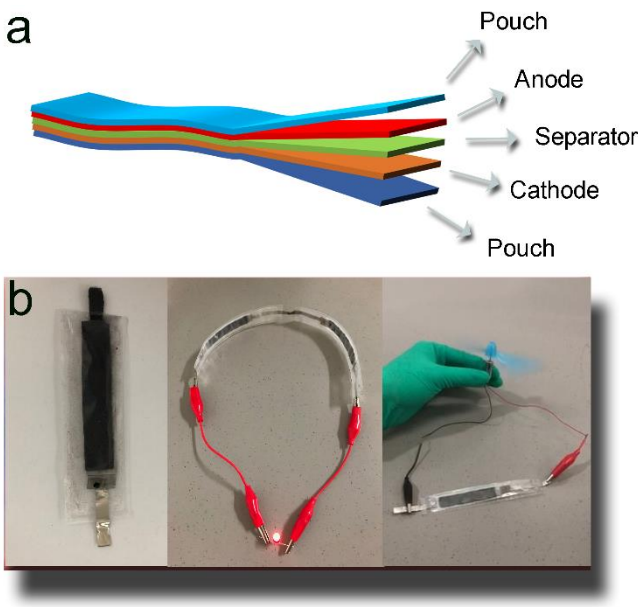

Supplementary Figure 17 | Structure and practical demonstration of the flexible PTO//MnO_2@GF hydronium-ion battery. **a**, Schematic illustration of the “sandwich” configuration flexible PTO//MnO_2@GF battery. **b**, Optical photographs of the flexible belt-shaped batteries and demonstrations of practical powering a LED light and motivating an electric fan using one such battery, presenting stable voltage output.

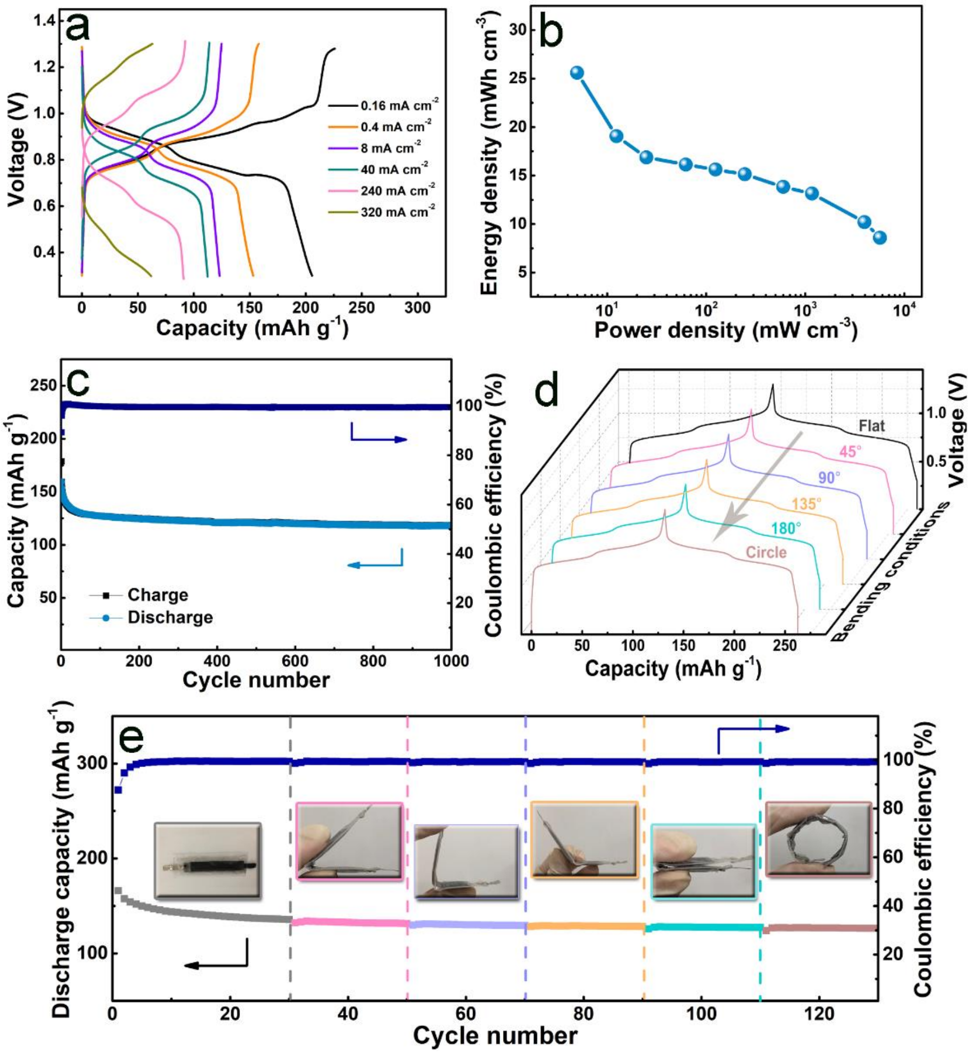

Supplementary Figure 18 | Electrochemical performance and flexibility property investigations of the flexible belt-shaped PTO//MnO_2@GF hydronium-ion battery. **a**, Rate ability described by galvanostatic charge-discharge profiles at various current densities. **b**, Ragone plot (i.e. volumetric energy density ~ power density). **c**, Cycle life examination at a current density of 2 mA cm^{-2} of the belt-shaped battery. **d**,**e**, Flexibility investigations by bending the belt-shaped battery to different degrees (i.e. 45°, 90°, 135°, 180°, bend to a circle, inspecting the galvanostatic charge-discharge curves (**d**) and cyclability (**e**) tested at a current density of 2 mA cm^{-2} under bending conditions.

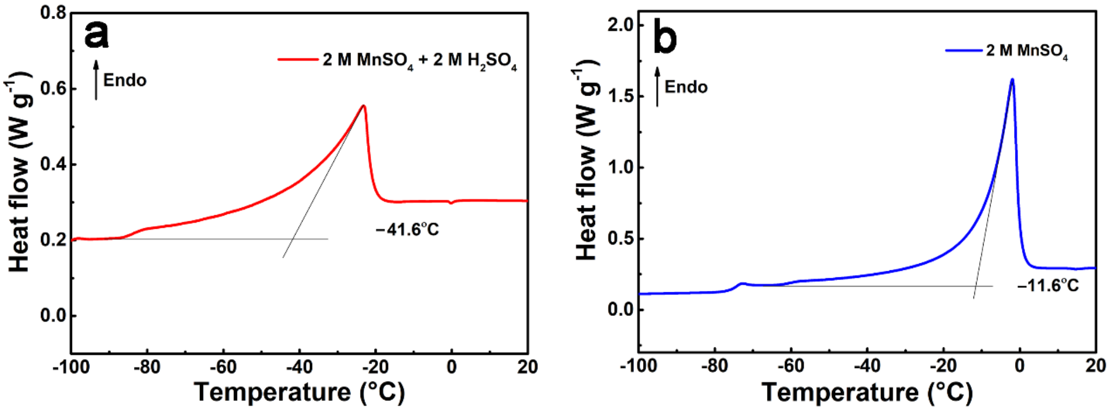

Supplementary Figure 19 | Differential scanning calorimetry (DSC) measurement to determinate the freezing point of electrolyte. **a**, 2 mol L^{-1} $MnSO_4$ + 2 mol L^{-1} H_2SO_4 solution. **b**, 2 mol L^{-1} $MnSO_4$ solution. [The freezing point of 2 M $MnSO_4$ + 2 M H_2SO_4 electrolyte and 2 M $MnSO_4$ electrolyte is determined as approximately -41.6 ^oC and -11.6 ^oC, respectively, which is derived from the onset melting point of the endothermal peak.]

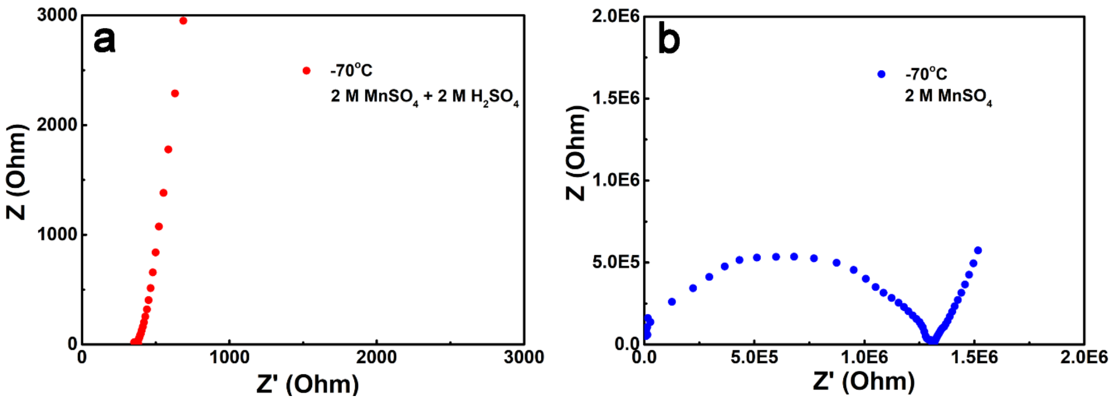

Supplementary Figure 20 | Nyquist plots of electrolytes at -70 °C to obtain the ion conductivity. **a**, 2 mol L^{-1} $MnSO_4$ + 2 mol L^{-1} H_2SO_4 solution. **b**, 2 mol L^{-1} $MnSO_4$ solution.

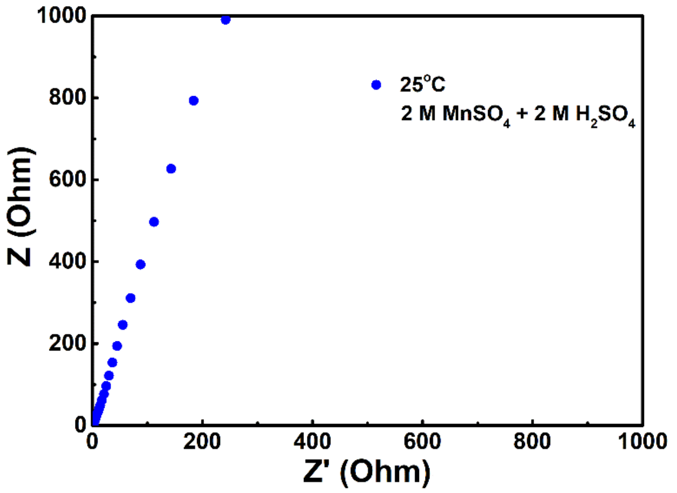

Supplementary Figure 21 | Nyquist plot of 2 mol L^{-1} $MnSO_4$ + 2 mol L^{-1} H_2SO_4 solution at room temperature (25 °C).

Supplementary Tables

Supplementary Table 1 | Comparison of energy and power densities between the previously proposed proton-based batteries/capacitors and this work.

Systems	Average Voltage (V)	Energy Density (Wh kg^{-1})	Power Density (mW kg^{-1})	Refs.
RuNH-OH/ RuCH-OH<br>Aqueous proton capacitor	0.4	1.84	311	S1
AQ//TCHQ<br>Aqueous proton capacitor	0.6	20.3	1.96	S2
Ru(II,III) complexes<br>Organic proton battery	0.2	3.4	2.3	S3
PEDOT-AQ//PEDOT-BQ<br>Organic proton battery	0.5	6.16	0.4	S4
$WO_3 \cdot 0.6H_2O$<br>Aqueous proton-storage electrode	-0.32	28.8	3.2	S5
**PTO//MnO_2@GF**<br>**Aqueous hydronium-ion battery**	**0.85**	**132.6**	**30.8**	**This work**

Note: The energy and power densities of systems in refs. S1 - S4 and this work are calculated based on the total weight of active materials of anode and cathode. While the energy and power densities are calculated based on the weight of active material of $WO_3 \cdot 0.6H_2O$ electrode for ref. S5. It should be noted that when converted into practical devices, a great discount should be considered for these calculated values because of the additional materials of a practical device, including current collector, separator, electrolyte, package and so on.

Supplementary Table 2 | Comparison of volumetric energy and power densities between the existed flexible energy storage systems (supercapacitors & batteries) and this work.

Flexible Systems	Electrode-active Materials	Energy Density ($mWh\ cm^{-3}$)	Power Density ($W\ cm^{-3}$)	Refs.
Super-capacitors	ppy-MnO_2-CF	6.16	0.4	S6
	Carbon/MnO_2 fibers	0.22	0.4	S7
	H-TiO_2@MnO_2//H-TiO_2@C	0.30	0.23	S8
	MnO_2//Fe_2O_3	0.55	0.139	S9
	HZnO@MnO_2	0.04	0.0024	S10
	MWCNT//$Li_4Ti_5O_{12}$	3.85	0.565	S11
	WO_{3-x}/MoO_{3-x}//PANI	1.9	0.73	S12
	Graphite/PANI	0.32	0.054	S13
	Co_3O_4//graphene	0.62	1.47	S14
	VO_x//VN	0.61	0.85	S15
	MWCNT/CMF	0.14	0.0027	S16
Batteries	$Na_{0.44}MnO_2$//$NaTi_2(PO_4)_3$	23.8	3.8	S17
	$Li_{1.1}Mn_2O_4$//$LiTi_2(PO_4)_3$	124	11.1	S18
	PTO//Zn	38.72	1.70	S19
	Zn//MnO_2	33.95	0.04	S20
	$LiMn_2O_4$//$Li_4Ti_5O_{12}$	10	—	S21
	$LiCoO_2$//LiPON//Li	2.2	—	S22
	**PTO//MnO_2@GF battery**	**25.7**	**5.67**	**This work**

Supplementary Table 3 | Performance comparison between the existed low-temperature (T) batteries and this work.

Battery Systems	Capacity/ Energy Density at Low-T	Relative Capacity/ Energy Density of 25°C	Low-T	Refs.
$NaTi_2(PO_4)_3$ ‖ 2 M $NaClO_4$-0.3 DMSO ‖ AC	68 mAh g^{-1} at 0.5 C (1 C = 133 mAh g^{-1})	61%	- 50°C	S23
$LiTi_2(PO_4)_3$ ‖ 0.5 M Li_2SO_4-0.3 DMSO ‖ AC	65 mAh g^{-1} at 0.5 C (1 C = 130 mAh g^{-1})	62%	- 50°C	S23
Polyimide ‖ 1M KCl-0.3 DMSO ‖ AC	58 mAh g^{-1} at 0.5 C (1 C = 183 mAh g^{-1})	62%	- 50°C	S23
PTPAn ‖ EA-based ‖ PNTCDA	69 mAh g^{-1} at 0.5 C (1 C = 100 mAh g^{-1})	70%	- 70°C	S24
Li ‖ Difluoromethane-based liquefied gas ‖ $LiCoO_2$	80 mAh g^{-1} at 0.2 C (1 C = 137 mAh g^{-1})	60.6%	- 60°C	S25
Li ‖ $LiPF_6$-EC/EMC ‖ NCM523 Self-heated battery	102 Wh kg^{-1} at 1 C (1 C = 7 A)	60%	- 40°C	S26
**PTO//MnO_2@GF hydronium-ion battery**	**110 mAh g^{-1} at 0.5 C (1 C = 200 mAh g^{-1})**	**73%**	**- 70°C**	**This work**

Supplementary Notes

Supplementary Note 1 | Morphology investigations of MnO_2@GF cathode undergoing various MnO_2 electrodeposition capacities on GF.

Nanosized-MnO_2 particles gradually increase on the surface of GF along with the electrodeposition time, and a compact MnO_2 layer forms after being charged for 1 hr (i.e. 5 mAh MnO_2). However, the MnO_2 layer begins to exfoliate under subsequent electrodeposition due to excessively thick of MnO_2 layer (**Supplementary Figure 12d**). Accordingly, the MnO_2@GF cathode is optimized to be prepared by pre-charging GF fibers for 1 hr at a current density of 5 mA cm^{-2} (i.e. 5 mAh MnO_2 electrodeposition capacity) for fabricating the PTO//MnO_2@GF full battery.

Supplementary Note 2 | Working mechanism on the cathode

It is well known that the discharge of MnO_2 in acid includes the ion intercalation (electrochemical process) and the disproportionated reaction of Mn^{3+} (chemical process), through which MnO_2 is converted into Mn^{2+}. Generally, the discharge can be summarized as:

Pathway-1: $2MnO_2 + 2H^+ + 2e^- \rightarrow 2MnOOH$ (electrochemical process) (1)

$2MnOOH + 2H^+ \rightarrow Mn^{2+} + MnO_2 + 2H_2O$ (chemical disproportionated reaction) (2)

Pathway-2: $MnO_2 + H^+ + e^- \rightarrow MnOOH$ (electrochemical process) (3)

$MnOOH + 3H^+ + e^- \rightarrow Mn^{2+} + 2H_2O$ (electrochemical process) (4)

The overall reaction of Pathway-1 and Pathway-2 is the same, which can be given as:

$$MnO_2 + 4H^+ + 2e^- \rightarrow Mn^{2+} + 2H_2O \quad (5)$$

Supplementary Note 3 | Calculations about energy density and power density of the (flexible) PTO//MnO_2@GF battery

The detailed calculations about the mass/volumetric energy and power densities of the (flexible) PTO//MnO_2@GF battery are given as follows:

(1) The specific energy density of the PTO//MnO_2@GF battery is calculated according to:

$$E_{spe} = \frac{C \times V}{1000 \times m} \qquad \text{Equation (1)}$$

where E_{spe} is the specific energy density (Wh kg^{-1}); C is the discharge capacity (mAh); V is the average discharge voltage (V); m is the total mass (kg) of anode (PTO) and cathode (the deposited or dissolved MnO_2; also see the discussion in **Supplementary Note 4**).

The specific power density of the PTO//MnO_2@GF battery is calculated based on:

$$P_{spe} = \frac{i \times V}{1000 \times m} \qquad \text{Equation (2)}$$

where P_{spe} is the specific power density (W kg^{-1}); i is the applied current (mA); V is the average discharge voltage (V); m is the total mass (kg) of anode (PTO) and cathode (the deposited or dissolved MnO_2).

(2) The volumetric energy density of the flexible PTO//MnO_2@GF battery is calculated according to:

$$E_{vol} = \frac{C \times V}{v} \qquad \text{Equation (3)}$$

where E_{vol} is the volumetric energy density (mWh cm^{-3}); C is the discharge capacity (mAh); V is the average discharge voltage (V); v is the volume (cm^3) of the flexible PTO//MnO_2@GF battery (1 cm x 5 cm x 0.04 cm).

The volumetric power density of the flexible PTO//MnO_2@GF battery is calculated according to:

$$P_{vol} = \frac{i \times V}{v} \qquad \text{Equation (4)}$$

where P_{vol} is the volumetric power density (mW cm^{-3}); i is the applied current (mA); V is the average discharge voltage (V); v is the volume (cm^3) of the flexible PTO//MnO_2@GF battery (1 cm x 5 cm x 0.04 cm).

Supplementary Note 4 | The hydronium-ion battery with high mass loading (25 mg cm^{-2} PTO)

The loading of MnO_2 depends on the charge depth. According to $It = nZF$, 1 mAh cm^{-2} charge capacity indicates a typical mass loading of 1.6 mg cm^{-2}. It is undoubted that the cathode and anode in a battery should keep capacity balance. Therefore, we control the charge depth (or mass loading) of MnO_2 by the mass of organic anode (that is PTO). In this work (**Figure 4**), the mass loading of PTO in anode is 4 mg cm^{-2}, which shows a capacity of 0.832 mAh cm^{-2} (= 4× 10^{-3} g cm^{-2} × 208 mAh g^{-1}). According to this value (0.832 mAh cm^{-2}), the loading of MnO_2 in cathode over a charge/discharge cycle is 1.33 mg cm^{-2}. Certainly, we can increase the loading MnO_2 in cathode by enhancing the mass loading PTO in anode. To clarify this point, we fabricated a battery using a much higher mass loading (25 mg cm^{-2}) of PTO in anode (as shown in **Supplementary Figure 16**). A capacity of 186.5 mAh g^{-1} could be achieved of the full battery with a high mass loading of active materials (PTO 25 mg cm^{-2}, i.e. MnO_2 of 8 mg cm^{-2}) at a low current density of 0.16 mA cm^{-2} (0.2 C). The reduced capacity of the hydronium-ion battery compared with that with mass loading of 4 mg cm^{-2} PTO should be attributable to the incomplete utilization of active materials due to the much higher active material loading.

Supplementary Note 5 | Electrochemical performance and flexible property of the flexible battery

The flexible belt-shaped PTO//MnO_2@GF hydronium-ion battery exhibits a remarkable rate ability with a respectable capacity of 89 mAh g^{-1} at an ultra-high rate (charged within 10 s, at a current density of 240 mA cm^{-2}, namely 300 C) (**Supplementary Figure 18a**). Taking the size of the whole battery (1 cm x 5 cm x 0.04 cm) into account, the belt-shaped battery presents a volumetric energy density up to 25.7 mWh cm^{-3} at a power density of 5.0 mW cm^{-3} and a volumetric power density of 5.67 W cm^{-3} at an energy density of 8.3 mWh cm^{-3} (**Supplementary Figure 18b**), much higher than most of the previously proposed flexible batteries and supercapacitors (see **Supplementary Table 2** for detailed comparison). Moreover, the belt-shaped battery achieves an extended cycling stability over 1000 cycles at a current density of 2 mA cm^{-2}, with a capacity retention of 78% and negligible decrease after 50 cycles (**Supplementary Figure 18c**). The robust mechanical property and bending-resistant ability was verified by exposing the battery to various deformation situations. Through successively changing bending degrees (45°, 90°, 135°, 180°, bent to a circle), the charge/discharge process of the belt-shaped battery exhibits sturdy performance with negligible capacity decay compared with the pristine unbending condition (**Supplementary Figure 18d**). It can be detected from **Supplementary Figure 18e** that even undergoing several charge-discharge cycles retaining each bending state, the specific capacity of the flexible prototype battery hardly presents attenuation.

Supplementary Note 6 | Ion conductivity of electrolytes at -70 °C

Calculated based on Nyquist plots in **Supplementary Figure 20**, the 2 M $MnSO_4$ +2 M H_2SO_4 electrolyte and 2 M $MnSO_4$ electrolyte show the conductivity of 2.66×10^{-3} S cm^{-1} and 7.962×10^{-7} S cm^{-1}, respectively, at -70 ^oC.

Supplementary Note 7 | Ion conductivity of electrolyte at room temperature (25 °C)

Calculated based on Nyquist plot in **Supplementary Figure 21**, the 2 M $MnSO_4$ +2 M H_2SO_4 electrolyte shows the conductivity of 5.31 × 10^{-1} S cm^{-1} at room temperature (25 °C).

Supplementary References

1. Yoshikawa, K. *et al.* Proton-rocking-chair-type redox capacitors based on indium tin oxide electrodes with multilayer films containing Ru complexes. *ACS Appl. Mater. Interfaces* **10**, 26990-27000 (2018).
2. Tomai, T. Mitani, S. Komatsu, D. Kawaguchi, Y. & Honma, I. Metal-free aqueous redox capacitor via proton rocking-chair system in an organic-based couple. *Sci. Rep.* **4**, 3591 (2014).
3. Motoyama, D. Yoshikawa, K. Ozawa, H. Tadokoro, M. & Haga, M. Energy-storage applications for a pH gradient between two benzimidazole-ligated ruthenium complexes that engage in proton-coupled electron-transfer reactions in solution. *Inorg. Chem.* **56**, 6419-6428 (2017).
4. Emanuelsson, R. Sterby, M. Stromme, M. & Sjodin, M. An all-organic proton battery. *J. Am. Chem. Soc.* **139**, 4828-4834 (2017).
5. Jiang, H. *et al.* Insights on the proton insertion mechanism in the electrode of hexagonal tungsten oxide hydrate. *J. Am. Chem. Soc.* **140**, 11556-11559 (2018).
6. Tao, J. *et al*. Solid-state high performance flexible supercapacitors based on polypyrrole-MnO_2-carbon fiber hybrid structure, *Sci. Rep.* **3**, 2286 (2013).
7. Xiao, X. *et al*. Fiber-based all-solid-state flexible supercapacitors for self-powered systems. *ACS Nano* **6**, 9200-9206 (2012).
8. Lu, X. *et al*. H-TiO_2@MnO_2//H-TiO_2@C core–shell nanowires for high performance and flexible asymmetric supercapacitors. *Adv. Mater.* **25**, 267-272 (2013).
9. Yang, P. *et al*. Low-cost high-performance solid-state asymmetric supercapacitors based on MnO_2 nanowires and Fe_2O_3 nanotubes. *Nano Lett.* **14**, 731-736 (2014).
10. Yang, P. *et al*. Hydrogenated ZnO core-shell nanocables for flexible supercapacitors and self-powered systems. *ACS Nano* **7**, 2617-2626 (2013).
11. Xiao, X. *et al*. Directly grown nanostructured electrodes for high volumetric energy density binder-free hybrid supercapacitors: a case study of CNTs//$Li_4Ti_5O_{12}$. *Sci. Rep.* **5**, 7780 (2015).
12. Xiao, X. *et al*. WO_{3-x}/MoO_{3-x} core/shell nanowires on carbon fabric as an anode for all-solid-state asymmetric supercapacitors. *Adv. Energy Mater.* **2**, 1328-1332 (2012).
13. Yao, B. *et al*. Paper-based solid-state supercapacitors with pencil-drawing graphite/polyaniline networks hybrid electrodes. *Nano Energy* **2**, 1071-1078 (2013).
14. Wang, X. *et al*. Fiber-based flexible all-solid-state asymmetric supercapacitors for integrated photodetecting system. *Angew. Chem. Int. Ed.* **53**, 1849-1853 (2014).
15. Lu, X. *et al*. High energy density asymmetric quasi-solid-state supercapacitor based on porous vanadium nitride nanowire anode. *Nano Lett.* **13**, 2628-2633 (2013).
16. Le, V. T. *et al*. Coaxial fiber supercapacitor using all-carbon material electrodes. *ACS Nano* **7**, 5940-5947 (2013).
17. Guo, Z. *et al*. Multi-functional flexible aqueous sodium-ion batteries with high safety. *Chem* **3**, 348-362 (2017).
18. Dong, X. Chen, L. Su, X. Wang, Y. & Xia, Y. Flexible aqueous lithium-ion battery with high safety and large volumetric energy density. *Angew. Chem. Int. Ed.* **53**, 7474-7477 (2016).
19. Guo, Z. *et al.* An environmentally friendly and flexible aqueous zinc battery using an organic cathode. *Angew. Chem. Int. Ed.* **57**, 11737-11741 (2018).

20. Zeng, Y. *et al*. Achieving ultrahigh energy density and long durability in a flexible rechargeable quasi-solid-state Zn-MnO_2 battery. *Adv. Mater.* **29**, 1700274 (2017).
21. Yang, Y. *et al*. Transparent lithium-ion batteries. *PNAS* **108**, 13013-13018 (2011).
22. Koo, M. *et al*. Bendable inorganic thin-film battery for fully flexible electronic systems. *Nano Lett.* **12**, 4810-4816 (2012).
23. Nian, Q. *et al*. Aqueous batteries operated at -50°C. *Angew. Chem. Int. Ed.* **58**, 16994-16999 (2019).
24. Dong, X. Guo, Z. Guo, Z. Wang, Y. & Xia, Y. Organic batteries operated at -70°C. *Joule* **2**, 902-913 (2018).
25. Rustomji, C. *et al*. Liquefied gas electrolytes for electrochemical energy storage devices. *Science* **356**, 1351 (2017).
26. Wang, C. *et al*. Lithium-ion battery structure that self-heats at low temperatures. *Nature* **529**, 515 (2016).
