# Supplementary material for: An organic/inorganic electrode-based hydronium-ion battery
Source: Nat Commun. 2020 Feb 19;11:959. doi: 10.1038/s41467-020-14748-5 (PMC7031366; doi:10.1038/s41467-020-14748-5)
Supplement: Supplementary file 3 — Description of Additional Supplementary Files [file 41467_2020_14748_MOESM3_ESM.pdf]

Description of Additional Supplementary Files

File Name: Supplementary Movie 1
Description: A short movie showing the details of low-temperature tests of the battery at -70°C.
